# Pathological Anatomy

**Published:** 1861-09

**Authors:** John H. Packard

**Affiliations:** Philadelphia


### PATHOLOGICAL ANATOMY.

BY JOHN H. PACKARD, M.D., OF PHILADELPHIA.

I.—*Hemorrhage into the Corpus Callosum.* Reported by Dr. HALDANE to the Medico-Chirurgical Society of Edinburgh.

A man about 60 years of age, of intemperate habits, fell down some steps and struck his head against a door. He was brought into the hospital in a state of concussion, from which he did not recover, but died about twenty-four hours after admission. On post-mortem examination a scalp wound, extending down to the bone, was found behind the right ear. There was no fracture of the cranial bones. When the brain was removed, a few drops of blood were found lying on the dura mater which lines the middle fossa of the skull on the right side. On slicing the brain, two or three small hemorrhagic extravasations, about the size of pepper-corns, were found in the substance of the corpus callosum. There was a small superficial clot in the left corpus striatum, and a similar lesion was found in each optic thalamus. Dr. Haldane remarked that hemorrhage into the corpus callosum was, as an idiopathic lesion, almost if not quite unknown; while, even as the result of injury, it was very rare, except in cases where there was extensive disorganization of the brain. In the present case the different hemorrhagic extravasations had no doubt been the result of injury, but they were extremely limited in extent; and it was certainly remarkable that a comparatively non-vascular part, like the corpus callosum, should be one of the seats of the lesion. The material lesions of the brain did not seem to be sufficient to account for the fatal result; in fact, as indicated by the symptoms observed during life, the individual died from concussion, having never recovered from the shock of the fall.—(*Dublin Medical Press*, May 15, 1861.)

II.—*Fibro-nucleated Tumor of the Brain.* Reported by Dr. SANDS to the New York Pathological Society.

A barkeeper, aged 37, who had been in his usual health on retiring for the night, was found at five o'clock in the morning trying to tear off his night-shirt, and saying that he was poisoned. When seen by Dr. Sands he was dying; perfectly insensible, his respiration nearly extinct, his pulse 120, his pupils widely dilated. At the autopsy, besides slight abnormities of the heart and kidneys, easily accounted for by the intemperate habits of the deceased, there were some interesting lesions observed in the brain. The tuber annulare was increased in breadth by the presence within it of an apoplectic clot, to which death was probably owing. "The calvarium was removed in the usual manner; but when the dura mater was cut through on the left side by a scalpel, it was noticed to pass through a soft substance which was not brain matter. Care was then taken to see what was the trouble, when a tumor was discovered, developed from the arachnoid surface of the dura mater. This tumor was hemispherical in its shape, its flattened surface being its point of attachment, the convex surface crowding upon the surface of the hemisphere without showing any signs of inflammation in the vicinity. The greatest breadth of the tumor was about an inch and a half; its surface was finely lobular, and presented a very vascular appearance." Examined with the microscope the tumor was found to contain "a very appreciable amount of connective tissue, disposed in bands, running in various directions, many of them having a concentric direction inclosing circular spaces. These spaces appeared to be filled partly with nuclei and partly with cells." The tumor was thought by Dr. Sands to be "one of a very rare form, to which Mr. Bennett, the Edinburgh physician, has given the name of fibro-nucleated—a name adopted by Mr. Paget—and in his opinion the tumor is closely allied to those known as fibro-recurrent. The nuclei abound considerably above the cells; they are oval, and contain either one or two nuclei. Both the nuclei and nucleoli are very minute, the average long diameter of the former being $\frac{1}{3000}$ of an inch. The cells are exceedingly slender, and correspond in appearance with those found in the fibro-recurrent tumor. Notwithstanding the tumor seems to have existed a considerable length of time, there were, according to the account of one of his friends, no brain symptoms at any time present, excepting the occasional attacks of delirium tremens."

Several other cases were mentioned in illustration of the fact that tumors and abscesses of the brain sometimes are, and sometimes are not, attended by symptoms recognizable during life.—(*Amer. Med. Times*, June 22, 1861.)

III.—*Obscure Cases of Cancer of the Œsophagus.* By DR. WILKS.

When this disease causes stricture of the tube, and consequently dysphagia, its diagnosis is usually clear; but sometimes the difficulty of swallowing is masked by the loss of appetite and the rapidly-increasing exhaustion of the patient, and such cases may be extremely obscure. Dr. Wilks has several times seen the true nature of the lesion detected only upon post-mortem examinations. The two following cases were observed by him at Guy's Hospital:—

*Case* 1.—Cancer of the œsophagus pressing on the pulmonary artery, and simulating aneurism.—"William N., aged 30, a carter, was admitted on June 2, 1860. He had felt unwell for several months, but scarcely knew what ailed him. He made few complaints on admission, stating merely that he had a pain in his back, and was too unwell to work. On carefully examining his chest, a distinct prolonged bruit was heard with the systole of the heart over midsternum. The position was remarkable, as well as the sound, and suggested, among other conditions, an aneurism of the aorta. He was then asked if he had any difficulty in swallowing, and said that he had, although he never volunteered the statement, and even then did not lay much stress on the symptom, his only complaint being pain in the back, or rather in the neck, dysphagia never being alluded to. This bruit, which was prolonged over both the systole and diastole, but distinct from both, when taken in connection with the difficulty of swallowing, was supposed to indicate an aneurism. Thus he continued for some time, eating little, but never complaining of the effort to eat. He had much pain in the course of the cervical nerves on the right side. He subsequently became much worse, by implication of the right lung, and went home to die. Dr. Wilks hearing of his death shortly afterward, went to his residence to make the post-mortem examination. Both lungs were in the early stage of hepatization. In the middle part of the œsophagus there was a mass of cancer involving the lung on each side; the disease was much more extensive than usual, not only affecting the walls of the tube, but projecting in the form of a tolerably hard mass, the size of a small egg. This pressed on the right side of the heart, obstructing apparently both the tricuspid and pulmonary orifices; the latter especially was considerably compressed by the disease. The great difficulties of an examination in private precluded a very accurate observation of the relation of the parts; sufficient, however, was seen to afford an explanation of the bruit, and so of the mistaken diagnosis.

"*Case* 2.—Cancer of the œsophagus opening into the aorta.—Margaret H., aged 60, admitted April 17. On admission she appeared ill, but made no special complaint. She had worked hard, and appeared to be rather worn out than suffering from any positive disease. About a fortnight after admission she spat up a little blood, and this led to a further examination of the chest, but nothing could be discovered. Two days before death she spat up a little more blood, and at the time appeared very low. On the following day she again spat up some blood, and died.

"*Autopsy.*—The body was extremely blanched. There was extensive carcinoma of the middle part of the œsophagus, circumscribed above, but eating its way through the walls, and involving the lung on the left side. At each end the canal was somewhat constricted, but the diseased part formed a sacculus full of *débris* and coagulum. The cancer was seen as a white deposit, and outside was the hypertrophied muscular tissue. The walls were eaten away on the left side, so that on removing the left lung the diseased œsophagus was opened. At this part also it opened into the aorta by a hole of considerable size, having apparently proceeded along the bronchial artery and eaten off its origin. The inner coat of the aorta was detached from a softening process, and could be stripped off for a considerable distance from the spot. The adjacent lung was consolidated and softening, and in one part a sloughing cavity. Chronic pneumonia at the apex. The left ventricle closely contracted, as in hemorrhage. The stomach and duodenum were quite full of clot. There was a large coagulum modeled to the stomach, and serum above it. The intestine below contained blood."—(*Med. Times and Gazette*, June 15, 1861.)

IV.—*Malformation of the Heart, with Stricture of Pulmonary Artery.*

A boy, aged 7, was admitted into the Children's Hospital in Paris, suffering from cyanosis. He was pigeon-breasted, and his fingers and toes were clubbed. His pulse was small and irregular—90 to 100 in the minute; there was a distinct bruit de souffle in the carotids. His respiration was rapid, and he had two or three times daily an attack of extreme dyspnœa, with hard cough. His skin was cooler than natural, and he had frequent and severe headaches.

All his symptoms were gradually aggravated; he became very weak; his senses were dulled, his appetite failed, and diarrhœa came on. The immediate cause of his death was an attack of measles, the cough connected with which proved fatal on the eighth day.

At the autopsy, twenty-six hours after death, there was observed very little cadaveric rigidity. The skin was bluish, especially that of the extremities, lips, and nostrils; the lower limbs were œdematous, and all the veins were gorged with thick, dark blood.

"The veins of the meninges of the brain, the vertebral arteries, the internal carotids, were distended with blood of a violet color. The brain itself was firm, and, on cutting it by slices, the white substance was found studded with innumerable red points.

"The lungs were healthy, but small and collapsed. The thymus gland was very large. I do not remember having seen it so large, even in an infant born before time. The pericardium contained from an ounce and a half to two ounces of a yellow-colored serosity.

"*Heart.*—Walls thick, filled with black blood and fibrinous clots, as well as the origin of the large vessels. Having been removed and carefully measured, it yielded the following dimensions:—

| | |
|---|---|
| From the apex to the base of the heart . . . . . | 2·44 inches. |
| Transverse diameter . . . . . . . . . . . | 2·08 " |
| Circumference of ventricular portion at widest part . . | 29·92 " |
| From the uppermost part of the ventricle to the centre of the base anteriorly . . . . . . . . . . | 2·52 " |
| Ditto, posteriorly . . . . . . . . . . . . . | 2·12 " |
| Distance in a straight line between the right and left extremities . . . . . . . . . . | 1·97 " |

"There was no median furrow either anteriorly or posteriorly, nor was there any sloping of the upper part of the heart. There was found no interventricular septum. The thickness of the ventricular wall was considerable; the columnæ carneæ were very numerous and powerful.

"The pulmonary artery arose from the right extremity of the ventricle, with which it communicated by a narrow opening; the sigmoid valves presented nothing worthy of notice. The calibre of the artery was, however, less than usual. The aorta was dilated. It arose from the centre of the single ventricle, between the right auricle, which was much dilated, and the left auricle, which was rather contracted. The right auriculo-ventricular opening was larger than usual, but its valves presented neither ossification nor hardness. The arrangement of the venæ cavæ, and of the pulmonary vein, presented nothing worthy of note. The foramen of Botal was obliterated, as well as the ductus arteriosus."

In connection with this case the opinion is expressed that cyanosis is the result simply of a mixture of venous and arterial blood, and that it cannot be caused by any interference, temporary or permanent, with either circulatory system.—(*Boston Medical and Surgical Journal*, June 6, 1861; from *British-American Journal.*)

[The opinion above quoted is not in accordance, we think, with that of most pathologists in the United States. See a paper by Dr. Moreton Stillé, of Philadelphia, published in the *American Journal of the Med. Sciences* for July, 1844; also a very interesting case by Dr. J. F. Meigs, in the Proceedings of the Pathological Society of Philadelphia, in the same journal for October, 1860.—P.]

### V.—*Serous Cysts of the Breast.* By Drs. H. LENOX HODGE and J. J. WOODWARD.

Dr. Hodge presented, at a meeting of the Pathological Society of Philadelphia, a specimen, of which he gave the following history: "This breast was removed by Dr. Norris from a lady about thirty-five years of age. She first noticed a tumor in her right breast some seven years ago. It did not appear to increase much in size until last fall. It then began to enlarge, and at the time of the operation presented the appearance of an irregular tumor about two inches in length and breadth. Both breasts were small; the lady had been married for four years, but had never been pregnant. The whole gland was removed, as other cysts were probably scattered throughout it. During the operation the largest cyst was opened. It contained an ounce or two of thin serous fluid. There were found to be some ten cysts, all apparently situated in the midst of the tissue of the gland. They varied much in size, the smallest being about as large as the little beads of children's toys. They were provided with a distinct membrane, and could be readily turned out entire from their bed of fibrous tissue. The contents of most of them consisted of a light-colored, thin serum, but in a few the fluid was of a deeper color, and much more glutinous."

Dr. Woodward, reporting on an examination made by him of a section of the growth, spoke of the rarity of simple cysts of the mammary gland; those connected with fibrous growths or with cancer being much more common. Their anatomy, as studied by him, was as follows: "Imbedded in the morsel of gland tissue, was what at first appeared to be a single cyst, but which more exact observation showed to be three cysts, the largest about the size of a small cherry, and the smallest that of a pea. They were in the closest juxtaposition, and only separated by the thinnest septa.

"On puncturing the largest of these cysts, a *clear, yellowish fluid* escaped, which became slightly turbid on boiling, showing the presence of a *very small* quantity of albumen. Repeated examinations showed no forms in this liquid, except a few minute oil globules. The *walls* of this cyst were vascular, and composed chiefly of well-developed white fibrous tissue, in which were imbedded a considerable number of more or less atrophied fat-cells, in which the *nucleus* and a number of drops of diverse size, composed of high-colored fat, could be distinguished readily.

"*The fluid of the second cyst* was more viscid, coagulated more abundantly, and therefore contained more albumen than the first. It also contained large numbers of large, pale cells, with delicate contours and dimly granular contents, $\frac{1}{150}$ to $\frac{1}{800}$ of an inch in diameter, and each containing a clear nucleus of from $\frac{1}{3300}$ to $\frac{1}{2500}$ of an inch in diameter. Some of these cells were partly destroyed, and the nucleus set free, with or without a portion of the contents adhering to it. They were sometimes adherent by their edges, and were evidently derived from the *distinct pavement-epithelium* with which the cyst was lined. This consisted of tolerably regular hexagonal cells, rather smaller and more granular than the bloated ones floating in the fluid, viz.: $\frac{1}{1200}$ to $\frac{1}{1000}$ of an inch in diameter, with nuclei $\frac{1}{5000}$ to $\frac{1}{3000}$ of an inch. The epithelium reposed on a fibrous layer, constituting the wall of the cyst, and like that of the first, vascular, and containing atrophying fat-cells. The third cyst was not examined microscopically. The tissue of the mammary gland was, in every respect, perfectly normal, so far as examined; the gland-vesicles presenting the usual characteristics. Groups of gland-vesicles, with the accompanying milk ducts, also perfectly healthy, were imbedded in the outer layers of the walls of the cyst.

"With regard to the origin of mammary cysts in general," Dr. W. thought "any exclusive doctrine must inevitably fall to the ground in the presence of individual cases. Mammary cysts may undoubtedly arise by the distention of one or more of the ultimate gland-lobules; perhaps even a lactiferous duct may dilate into a cyst in consequence of obstruction. But besides these methods, by which the gland itself might contribute to cyst-formation, cysts may arise in the areolar tissue connecting the lobules together, by a dilatation of one or more of the areolæ. Rokitansky's idea, that cysts are special hetorologous products, which begin as microscopic nuclei and dilate into cysts, had not met with general credence.

"Besides the theories of cyst-development with which he was familiar, one had suggested itself to him which he considered worthy of mention, and that was the possibility of the formation of cysts, by a process of *vacuolation*, in an intercellular matrix, similar to that by which the areolæ of areolar tissue, the intercellular passages in leaves, and perhaps all the closed cavities of the body, are developed.

"He had seen in the villi of the chorion, in a case of so-called 'hydatid mole,' microscopic cysts which appeared to be merely cavities formed by the solution of the jelly-like intercellular tissue, pushing the cells aside, and limited apparently by no distinct wall other than the substance in which they were excavated.

"In the mammary cysts above described, he believed the presence of the atrophying fat-cells in the walls was sufficient to show that the tissue of the cyst-wall was not of new formation, but represented the normal connective tissue of the gland, pushed aside, and distended by the accumulation of the liquid. The cyst described as lined by the epithelium he was disposed to regard as having originated in the dilatation of one or more of the gland-lobules—the epithelium being furnished by the overgrowth of the secreting cells of the lobule. The first cyst, which was larger, might represent one of similar origin in which the epithelium had perished; or perhaps it had originated in the connective tissue between the lobules, and not in these themselves."—(*Proceedings of the Pathological Society of Philadelphia*, in the *Am. Journal of the Med. Sciences*, April, 1861.)

VI.—*Congenital Absence of the Gall-bladder.* Reported by DR. ALEXANDER R. SIMPSON to the Edinburgh Medico-Chirurgical Society.

A child about six weeks old died, having been affected with what seemed to be sclerema of the face, arms, and legs; there was no albuminuria. "On making a post-mortem examination, the skin was found to be intensely indurated in patches all over the legs, hips, arms, and cheeks; and when incisions were made into these parts, there flowed out a pale, faintly straw-colored fluid, which, on standing, presented a fibrinous coagulum. A small orifice yielding pus was noticed on the middle of the back, opposite the spine of the ninth dorsal vertebra, which was found, on passing a probe through it, to communicate with a large cavity underneath the skin, of a lozenge shape, and extending in a longitudinal direction from a point opposite the spine of the fifth dorsal vertebra above, to another opposite the fourth lumbar vertebra below, and laterally between the levels of the angles of the ribs. In this extended space the subcutaneous cellular tissue was all destroyed, only some fibrous bands, containing vessels and nerves, still connecting the skin with the aponeurosis of the back. The thoracic organs were perfectly healthy and natural; the lungs everywhere fully distended and filled with air. The abdomen contained about a teacupful of yellow serum, intermingled with fibrinous flocculi. The intestines were injected on the surface, of a pale roseate or pink color, and matted together at parts with fibrinous deposits. These fibrinous exudations were also seen on the surface of the pelvic organs, as well as of the liver and spleen. The spleen and kidneys were healthy. No albumen could be detected in the few drops of water present in the bladder. The gall-bladder being 'conspicuous by its absence' from the lower surface of the liver, that organ was carefully removed, along with the spleen, stomach, duodenum, and diaphragm. It was found to be almost incased by a thick, yellowish layer of fibrin, by means of which it was loosely bound to the diaphragm. As members of the Society could observe, there was no trace of the existence of a gall-bladder on the lower surface of the liver, nor was there any depression corresponding to its usual site. The left lobe of the liver was nearly of equal size with the right; and although on the latter there was a faint attempt at the formation of a lobulus quadratus, there was no lobulus Spigelii or lobulus caudatus whatever. The right and left lobes were united by a thick pons hepatis over the corner of the ductus venosus, so that the latter appeared to penetrate the substance of the liver. On laying open the duodenum, the orifice of the bile-duct was at once seen in its ordinary situation, and a single drop of pale bile could be expressed from it. On tracing this duct to the liver, it was found to pass up undivided into the horizontal fissure, where it broke up at once into the hepatic tissue of the right and left lobes." — (*Dublin Medical Press*, June 5, 1861; from *Edinburgh Medical Journal.*)

VII.—*Complete Transposition of all the Thoracic and Abdominal Viscera.* By EDWARD PARKER YOUNG, Esq., M.R.C.S.

A lady, aged eighty-five, who had, previously to her death, (with the exception of a slight cold,) enjoyed good health, was found dead in her room. A post-mortem examination being made, "on opening the thorax and abdomen, a complete transposition of all the organs presented itself. The heart lay with its base toward the left side of the spinal column, the apex pointing toward the right side and reaching to the lower border of the fourth rib under the right mamma. The venæ cavæ were situated on the left side, passing into the pulmonary cavity of the heart, which was also on the left side; the aorta and systemic ventricle to the right; so that not only was the heart reversed in position, but also in formation. The left phrenic vein was lying on the superior vena cava; the right innominata was seen passing over the aorta to the left, and emptying itself into the superior vena cava. The lungs were healthy, but old pleuritic adhesions existed on both right and left sides, especially the former. The larger lobe of the liver was in close proximity to the left ribs, the smaller lobe extending only slightly to the right of the sternum. The spleen was situated on the right side, just beneath the heart; œsophagus lying to the right of the aorta. The stomach was situated on the right side, the cardiac extremity touching the ribs, and the pyloric end extending to the left side of the mesial line; the sigmoid flexure of the colon was on the right side. The heart and its valves were healthy. The head was examined. On removing the calvaria, the veins over the surface of the brain were seen to be highly congested, and coagulated lymph was found beneath the arachnoid; the brain-substance was firm; the puncta vasculosa were more numerous and visible than usual; the lateral ventricles contained a small quantity of serum, and there was œdema of the choroid plexus, giving them the appearance of a small bunch of white currants. About two ounces of fluid were found at the base of the brain. No clot could be discovered, though a minute inspection was made."—(*Lancet*, June 29, 1861.)

